# CSACI position statement: Newer generation H_1_-antihistamines are safer than first-generation H_1_-antihistamines and should be the first-line antihistamines for the treatment of allergic rhinitis and urticaria

**DOI:** 10.1186/s13223-019-0375-9

**Published:** 2019-10-01

**Authors:** Michael N. Fein, David A. Fischer, Andrew W. O’Keefe, Gord L. Sussman

**Affiliations:** 1 Division of Clinical Immunology and Allergy, McGill University, Montreal, QC Canada; 2 Division of Allergy and Clinical Immunology, Western University, London, ON Canada; 3 The Canadian Society of Allergy and Clinical Immunology, Ottawa, ON Canada; 4 Division of Pediatrics, Faculty of Medicine, Memorial University, St. John’s, NL Canada; 5 Gordon Sussman Clinical Research, Toronto, ON Canada

**Keywords:** Histamine, H_1_-antihistamines, Diphenhydramine, CSACI position statement

## Abstract

Oral H_1_-antihistamines (AHs) are the most commonly used therapy to treat allergic rhinitis and chronic urticaria. Older, first-generation AHs (e.g. diphenhydramine, hydroxyzine) have significant and common side effects including sedation, impairment with decreased cognitive function, poor sleep quality, dry mouth, dizziness, and orthostatic hypotension. These drugs have also been found to result in death from accidents, intentional or unintentional overdoses, and sudden cardiac death. The unfavourable risk–benefit profile of first-generation AHs led to the development of newer, less-sedating second- and third-generation AHs, which first became available in Canada in the 1980s. High-quality trials have proven that newer generation AHs are superior in safety compared to older first-generation AHs. On average, they have improved potency and efficacy. Second- and third-generation AHs are the recommended first-line treatment for mild allergic rhinitis and acute and chronic urticaria. Despite this evidence, older first-generation AHs continue to be over-utilized because of their over-the-counter (OTC) status and long history of use. The Canadian Society of Allergy Clinical Immunology (CSACI) recommends that newer generation AHs should be preferred over first-generation AHs for the treatment of allergic rhino-conjunctivitis and urticaria. To promote this recommendation, education of health professionals and the public is necessary. Further, given the dangers of older first-generation AHs, we believe they should be used only as a last resort with eventual consideration given to having them only available behind the counter in pharmacies.

## Background

Histamine is a biogenic amine whose release results in allergic rhino-conjunctival symptoms as well as the urticarial wheal and flare reaction with itch [1].

H_1_-antihistamines are the most utilized class of medications for the treatment of allergic rhino-conjunctivitis and urticaria [2]. The most well-known first-generation AH, diphenhydramine (Benadryl©) has been available since 1946. This drug was introduced before current licensing standards, and thus it did not pass the rigorous safety and efficacy standards required today [1]. Since their introduction, the association between first-generation AHs and dangerous side effects, including sedation, respiratory depression, coma, and death, has become clear. However, because of their longevity, name recognition, and OTC status, both patients and practitioners continue to select older more dangerous first-generation AHs over well-studied newer, safer and affordable alternatives [3]. Furthermore, adverse events associated with these medications often preclude patients from trying other treatment options.

The unfavourable risk–benefit profile of first-generation AHs has led to the creation of newer, less-sedating 2nd- and 3rd-generation AHs, which first became available in Canada in the 1980s. Third-generation antihistamines are defined as being metabolites or enantiomers of previously available drugs and can therefore lead to an increase in efficacy and/or safety. In Canada these include: fexofenadine and desloratidine [4]. High-quality trials have proven newer generation AHs are superior in safety, are faster in onset of action, and have improved efficacy, length of action and potency compared to older 1st generation AHs [2, 5, 6]. Newer generation AHs are first-line treatment for mild allergic rhino-conjunctivitis and acute and chronic urticaria [7–9]. The cost of newer generation antihistamines has come down significantly and several are now available OTC. Despite the decrease in price, the newer generation AHs are often still more expensive than first generation AHs and cost remains a barrier.

Outside of North America, 1st generation antihistamines are mainly used for the treatment of motion sickness and as sleep aids, and not for the routine treatment of allergic rhinitis and urticaria because of their sedative tendencies. However, in Canada, online surveys of physicians and pharmacists show that Benadryl©(diphenhydramine) remains the most recommended antihistamine for allergic symptoms in children in each of the last 7 years [10]. The aim of this position statement is to highlight the known and newly recognized risks associated with first-generation AHs, to review the evidence of superior safety of newer generation AHs, and to recommend against the routine use of first-generation AHs (Table 1).

**Table 1:** H1 Antihistamines: pharmacokinetics and pharmacodynamics in healthy adults. Reproduced with permission [5]

Orally administered H1-antihistamines	Time to maximum plasma concentration (h) after a single dose	Terminal elimination half-life (h)	Clinically relevant drug–drug interactions^a^	Onset of action (h)^b^	Duration of action (h)^b^
First (old) generation					
Chlorpheniramine^c^	2.8 ± 0.8	27.9 ± 8.7	Possible	3	24
Diphenhydramine^c^	1.7 ± 1.0	9.2 ± 2.5	Possible	2	12
Doxepin^c^	2	13	Possible	NA	NA
Hydroxyzine^c^	2.1 ± 0.4	20 ± 4.0	Possible	2	24
Second (new) generation					
Bilastine	1.2	14.5	Unlikely	2	24
Cetrizine	1.0 ± 0.5	6.5–10	Unlikely	0.7	≥ 24
Desloratidine	1.0–3.0	27	Unlikely	2–2.6	≥ 24
Fexofenadine^a^	1.0–3.0	11.0–15.0	Unlikely	1.0–3.0	24
Levocetirizine	0.8 ± 0.5	7 ± 1.5	Unlikely	0.7	> 24
Loratidine (metabolite: descarboethoxyloratidine)	1.2 ± 0.3 (1.5 ± 0.7)	7.8 ± 4.2 (24 ± 9.8)	Unlikely	2	24
Rupatadine	0.75–1.0	6 (4.3–14.3)	Unlikely	2	24

^a^Clinically relevant drug–drug interactions are unlikely with most of the 2nd generation H1-antihistamines. Clinically relevant drug-food interactions have been well studied for fexofenadine. Naringin, a flavonoid found in grapefruit juice, and hesperidin, a flavonoid in orange juice, reduce the oral bioavailability of fexofenadine through the inhibition of OATP 1A2. This interaction can be avoided by waiting for 4 h between juice ingestion and fexofenadine dosing

^b^Onset/duration of action is based on wheal and flare studies

^c^Six or seven decades ago, when many of the first-generation H_1_-antihistamines were introduced, pharmacokinetic and pharmacodynamic studies were not required by regulatory agencies. They have subsequently been performed for some of these drugs; however, empiric dosage regimens persist. For example, the manufacturers’ recommended diphenhydramine dose for allergic rhinitis is 25 to 50 mg every 4 to 6 h, and the diphenhydramine dose for insomnia is 25 to 50 mg at bedtime. Despite the long terminal elimination half-life values identified for some of the medications (e.g., > 24 h for chlorpheniramine), based on tradition, extended release formulations remain in use

## Risks of first generation H1-antihistamines

Shortly after their introduction in the 1940s, the potential for severe adverse effects associated with their use was reported [11–13]. These older AHs have poor receptor selectivity and non-specifically bind muscarinic, serotonin, and α-adrenergic receptors, as well as cardiac potassium ion channels, leading to several intolerable and potentially life-threatening adverse effects [2]. They also cross the blood–brain barrier and may lead to significant CNS suppression and toxicity resulting in psychomotor impairment, coma, and even death [14]. Because of safety concerns, in 2009 Health Canada recommended that 1st Generation antihistamines not be sold in combination with other drugs to children under 6 for coughs and colds [15].

### CNS suppression: sedation, poor sleep quality and decreased cognitive performance

Older, first-generation AHs are commonly used as sleep aids because of their strong sedative qualities. Surprisingly, the dose utilized for sleep induction is the same dose used for rhinitis symptoms. Despite their sedative effects, these older medications do not result in quality sleep [16, 17]. After a night-time dose of chlorpheniramine (1st generation), next-day “hang-over” effects like impaired vigilance, divided attention, working memory, sensory-motor performance, and reduced latency to daytime sleep has been observed [17].

In addition to poor sleep quality and increased sedation, older AHs have also been associated with decreased school performance measures [17]. Walker et al. found that students with symptoms of allergic rhinitis were 40% more likely to drop a grade from practice tests to final examinations and 70% more likely to drop a grade if they reported taking older sedating AHs [3].

### CNS impairment and accidents

First-generation AHs have been associated with injuries and fatalities due to car, plane, and boating accidents [14]. A randomized controlled trial comparing fexofenadine 60 mg, diphenhydramine 50 mg, alcohol (0.1% blood alcohol concentration), and placebo found that driving performance was the *poorest after diphenhydramine use* and that drowsiness ratings were not predictive of the level of impairment [18]. Despite warnings that diphenhydramine may cause drowsiness and should not be taken when operating machinery it is not specified that this includes driving [19].

In a review of 484 fatalities in Ontario, drivers who were killed in car accidents and found to be at fault were 1.5 times more likely to have been under the influence of first-generation AHs [20]. The European Union has labelled diphenhydramine a Category III drug indicating that it is likely to produce severe effects on fitness to drive [21]. This carries their highest warning level and the recommendation: “Do not drive. Seek medical advice before driving again.” [21].

In a recent review of toxicology testing profiles from 6677 fatally injured civil aviation pilots in the United States from 1990 to 2012, diphenhydramine was the drug most commonly found on autopsy that was capable of causing impairment (7.3%) [22]. Due to the increased risk, first-generation AHs are banned for use by commercial and military airline pilots before or during flights [2, 5].

### Overdose and toxicity

Diphenhydramine and other first-generation AHs are documented drugs of abuse, and overdose can result in significant anti-cholinergic effects including fever, flushing, pupillary dilatation, urinary retention, tachycardia, hypotension and coma [23]. Infants and children who experience accidental or intentional overdose may present with paradoxical excitation including irritability, hallucinations, and seizures followed by drowsiness, delirium, respiratory depression and coma [14, 23, 24]. In 2003, 28,092 exposures to diphenhydramine were reported to poison control centres in the United States—11,355 (40.4%) of these cases were in children under the age of six, resulting in at least six fatalities [23].

### Risk of QT prolongation and torsade de pointes

Cardiac toxicity is an increasing concern with use of first-generation AHs, especially amongst older patients with significant comorbidities and polypharmacy via drug interactions.

The cardiac safety of first generation antihistamines was never studied as this was an unknown risk when introduced. In June 2016, Health Canada released a safety recall regarding hydroxyzine and issued a “black box” warning hydroxyzine can increase the risk of QT prolongation and torsade de pointes. Hydroxyzine has the potential to cause dizziness, palpitations, syncope, seizures, or sudden cardiac death [25, 26]. Furthermore, the new maximum daily dose has been reduced to 100 mg in adults and 50 mg in the elderly, if the medication cannot be entirely avoided.

## Newer generation H_1_-antihistamines

Second- and third-generation non-sedating AHs were developed with decreased ability to cross the blood–brain barrier and without anticholinergic effects. Initially available for clinical use since 1981, this growing class of medications has been extensively studied in high-quality, randomized controlled clinical trials [27–30]. These studies demonstrated safety even in off-label high-dose regimens [31]. They have also been found to have an equivalent or faster onset of action compared to first-generation AHs [2, 5, 32]. Jones et al. found that the time to induce a 50% reduction in histamine-induced flare response for oral diphenhydramine (50 mg) was 79.2 min [33]. In contrast, the same outcome took 50 min for cetirizine [34, 35].

### Superior safety of newer generation H_1_-antihistamines

Although not without side effects, in contrast to older generation AHs, newer generation medications have minimal serious safety concerns [5, 36]. There have been no fatalities directly associated with the use of the newer generation AHs available in Canada. Accidental exposures of up to 30-fold ingestions of cetirizine, loratadine, and fexofenadine have not resulted in any serious adverse events [37–39].

Two second-generation AHs (astemizole and terfenadine) have been associated with cardiac toxicity, however, both were removed from the market over 20 years ago. Since this time there have been no new concerns regarding cardiac toxicity and second- and third- generation AHs [5, 37]. Since the discovery of the above-noted cardiac toxicity, all second- and third-generation AHs are required by regulatory agencies to undergo thorough cardiac safety testing at standard and high off-label doses [5].

The level of sedation experienced by patients taking newer generation AHs varies by specific medication and the dose. Loratadine, fexofenadine, desloratidine, rupatadine and bilastine are the least-sedating antihistamines and, presently, loratadine, fexofenadine and desloratidine are the sanctioned choices for pilots, truck drivers and others who perform complex tasks like operating heavy machinery vs. cetirizine which is a low-sedating antihistamine [6, 9, 40].

### Efficacy of first generation antihistamines versus newer generation

Perceived quicker onset of action of older AHs is often cited as a reason why patients and practitioners choose first-generation medications, however this perception has been proven inaccurate in clinical studies [33, 41]. Indeed, in a double-blind placebo controlled trial, both cetirizine and loratadine were found to have significantly faster onset of action, potency, and duration of action when compared to chlorpheniramine [42]. The prolonged duration of action with newer generation antihistamines vs. 1st generation antihistamines is also a distinct advantage [6] Epinephrine is the drug of choice for anaphylaxis, but H1-antihistamines are also used in its treatment, with the route of treatment varying with severity of reaction. Only 1st generation antihistamines are available for IV use but they can potentially increase vasodilation and hypotension if given rapidly. If an oral antihistamine is to be given, a low sedating antihistamine like cetirizine, which is absorbed rapidly, is preferable to sedating antihistamines like diphenhydramine [43].

## Conclusion

First-generation AHs have been used for the treatment of allergic disease for over 70 years. However, common and serious adverse effects associated with these medications have been reported. First-generation AHs are in the process of being restricted as new evidence of harm, contraindications and dose limitations become apparent. Older AHs have not passed current safety or efficacy standards, and should not be used in routine circumstances for allergic disease. Newer generation AHs have been extensively studied over the past 30 years, and are safer, feature faster or equivalent onset of action, and are superior in efficacy compared to first generation AHs. The CSACI, therefore, recommends in agreement with other international bodies, that only less-sedating newer generation AHs should be first-line and preferred over older AHs and that the use of first-generation AHs should be significantly curtailed [9, 14].

### Key points

1. First-generation AHs are associated with significant and, at times, serious adverse effects including fatal outcomes, and they should not be used as first-line treatment in allergic disease.
2. Despite package warnings, the level of CNS impairment caused by first generation AHs is not fully appreciated both by health care professionals and the public, which has resulted in preventable fatal injuries.
3. Newer generation AHs are proven to be much safer than first-generation AHs, have a faster onset of action, and have superior potency, selectivity and efficacy.
4. Despite the widespread availability of newer generation AHs, older AHs remain over-utilized.
5. To encourage the cessation of the routine use of older AHs including diphenhydramine (Benadryl©), this class of medications should have eventual consideration for availability on a behind the counter basis only.
6. Further efforts are needed to disseminate this information to healthcare providers and patients to help change practice and improve patient health and safety.

## Data Availability

Not applicable.

## Abbreviation

AHs: antihistamines
